# Depression and anxiety in hospitalized children with epilepsy during COVID-19 pandemic: Preliminary findings of a cross-sectional study

**DOI:** 10.1192/j.eurpsy.2021.1780

**Published:** 2021-08-13

**Authors:** C. Correale, I. Tondo, C. Falamesca, T. Grimaldi Capitello, F. Vigevano, N. Specchio, S. Cappelletti

**Affiliations:** 1 Clinical Psychology, Bambino Gesù Children Hospital, Rome, Italy; 2 Neurological Sciences, Bambino Gesù Children Hospital, Rome, Italy

**Keywords:** COVID-19, Epilepsy, Anxiety, Depression

## Abstract

**Introduction:**

Anxious-depressive disorders are common among children with epilepsy. A recent Systematic Review and Meta-Analysis (Scott et al., 2020) reported that the overall pooled prevalence of anxiety disorders is 18.9% while of depressive disorders is 13.5%. COVID-19 pandemic has centralized the attention of governors and careers on the health emergency. As a result, the trajectory of the psychological care needs of this at risk population may have been neglected.

**Objectives:**

The aim of the study was to assess the prevalence rate of depressive and anxiety symptoms among children with epilepsy during COVID-19 pandemic. Children were hospitalized in- and out-patients under a neurological and psychological follow up program in an Italian Children Hospital.

**Methods:**

We conducted a cross-sectional study among 38 hospitalized children and adolescents with epilepsy (21F; 17M, mean age: 14,5; range: 11-18) during COVID-19 pandemic. We performed face-to face interviews and assessed depressive and anxiety symptoms with the Patient Health Questionnaire (PHQ-9) and the Generalized Anxiety Disorders (GAD-7) questionnaire during scheduled follow up checks.

**Results:**

Preliminary results showed a rate of mild-to-severe anxious depressive symptoms by 49.9% and 60.5% respectively. In detail: 21.1% mild, 15.7% moderate and 13.1% of severe anxiety, meanwhile 23.7% mild, 26.3% moderate and 10.5% of severe depression. The prevalence of comorbid depressive and anxiety symptoms was 39.5% among the entire sample.

**Conclusions:**

Depressive and anxiety rates among hospitalized children with epilepsy during COVID-19 outbreak are very high. Pediatric services should deserve special attention to those patients’ mental health. Regular screening protocols and empowerment interventions in Hospital should be promoted.

**Disclosure:**

No significant relationships.

